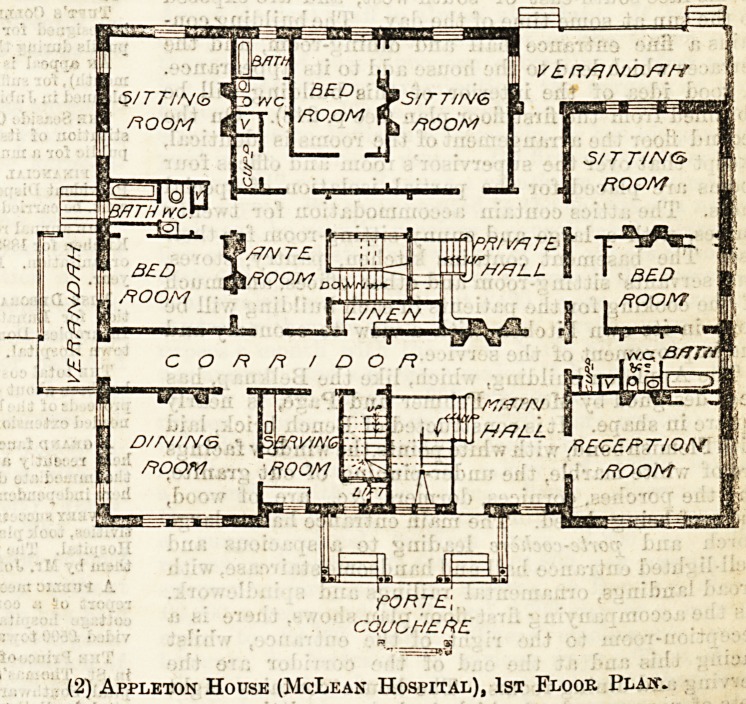# The McLean Hospital, Waverley, Mass., U.S.A.

**Published:** 1894-01-13

**Authors:** 


### The Institutional Workshop.

### HOSPITAL CONSTRUCTION.

### McLEAN HOSPITAL, WAVERLEY, MASS., U.S.A.

The construction of modern asylum buildings, where the superintendents are allowed a free hand, tends mainly in one direction. This direction is indicated by the circumstance that the McLean Lunatic Asylum, in recognition of the present broader views upon the subject of insanity and its treatment, is to be henceforth known in its new buildings as the McLean Hospital. The trustees point out that at its foundation the McLean Asylum rescued the unfortunate lunatic from a state of hopeless misery: it has given him with each succeeding year the larger help that medical skill and increasing resources in every direction have made available. Owing to the many disadvantages of its surroundings, the old site has been sold, and a new site has been purchased at Waverley, Mass., which is declared to be unsurpassed in suitable and attractive qualities. In determining the plan of construction the chief object held in view has been to secure that the McLean Hospital in its new home shall not only maintain the position which it has hitherto held, but bring "all the improvements of modern medicine to the diseased mind."

A, Administration House; B, Upham Memorial for Men; C, Belknap House for Men; D, Intermediate House for Men; E, Bowditch House for Men; F, Gymnasium for Men; G, Belknap House for Women; H, Appleton House for Women; I, Intermediate House for Women; J, Bowditch House for Women; K, Gymnasium for Women; L, Amusement Hall, Kitchen, and Laboratory; M, Superintendent's House; N, Pumping, Heating, and Electric Station; O, Stables; P, Gate Lodges; Q, Farm Cottages; R, Greenhouses.

As we have elsewhere pointed out, the older buildings especially, and in fact the majority of asylum buildings, are not well adapted to secure the most favourable results to the patient, owing to the absence of sufficient means of isolation and disconnection. Unfortunately most new asylums in the past have been but a reproduction in whole or in part of an existing building. The more closely the plans of the existing asylums are examined, the more evident does it become that up to the present time there has seldom been any departure, so far as construction is concerned, from the old types already referred to. Where changes have been introduced they have not gone to the root of the system with a view to offer facilities for treatment, and so to meet the requirements of advancing science and devoted care on the part of the medical superintendent. Dr. Bancroft, of the New Hampshire Asylum, Concord, U.S.A., in a paper read before the New England Psychological Society at Boston, indicated, with soberness and force, the directions in which alterations were desirable. Many of the most intelligent superintendents are fully alive to the facts adduced by Dr. Bancroft, and are, we believe, almost entirely in agreement with his views. Having charge of old buildings, they have set to work, as in the case of Dr. Clouston at the Morningside Asylum, Edinburgh, to reconstruct them bit by bit by breaking them up, and so making it possible to separate into groups patients who may be consigned to the larger wards. They have further caused cottages, detached houses, and mansions, like Craig House, Morningside, to be erected in various parts of the grounds, and have in this way gradually introduced, so far as circumstances have permitted, the system we are about to explain.

Dr. Cowles, of the McLean Asylum, who has devoted himself to the science of psychology and asylum management for very many years, has wisely endeavoured to make the removal from the old site the occasion to plan a hospital for the insane, so as to bring all the improvements of modern medicine and construction to aid the treatment of the patients committed to his care.

#### Hospitals for the Insane.

The State Legislature of New York has indicated that henceforth all institutions for the treatment of lunatics under its jurisdiction shall be denominated "Hospitals for the Insane." The trustees of the McLean Hospital for the Insane, though not under the jurisdiction of New

York State, have come to the same determination, because they rightly hold that it is important to impress on the public that lunacy is a disease which is often capable of treatment resulting in ultimate cure. At Waverley Dr. Cowles had a virgin site and a free hand. In planning the buildings and placing them on the site he has evidently been impressed, as Dr. Clouston was, with the importance of providing for the noisy class of patients separate from the others in blocks so situated as to be beyond the hearing of the quiet patients at all times. He has, further, broken the asylum up into a

Taking the new buildings as a whole, no one can fail to perceive that they have been planned and designed so as to lend themselves to the improvement, if anything, of the already beautiful site upon which they are placed. In order, no doubt, to secure as great a variety as possible, of the nine building serected, four, being the whole of the women's side, have been designed by Messrs. Fehmer and Page, two by Messrs. Shepley, Ruttan, and Coolidge, two by Messrs. Shore and Hunnewell, and one by Mr. W. Y. Peters. Thus eight architects have been employed to design nine

(1) Belknap House (McLean Hospital), 1st Floor Plan.

number of villas or mansions, situated on either side of the administration buildings, each of which will be constructed after a different design, and be so situated in the grounds as to give it an independent character. This system promotes a feeling of homeliness and comfort in the minds of the patients, which in many cases tends to hasten their recovery. Dr. Cowles, like Dr. Clouston and many other superintendents, has evidently taken a pride in thinking out and designing new features for every portion of the new McLean Hospital, the influence of which will extend to posterity, and the results of which must lead to incalculable good.

buildings, a circumstance unique in itself, and well calculated to emphasise the importance attached by the trustees of the McLean Hospital to the new departure in asylum construction, which the opening of the hospital at Waverley will celebrate.

We have thought it well to publish a plan of the site, and also the first floor plans of two of the buildings, namely, Belknap House and Appleton House, both occupied by women patients. Before describing these buildings in detail, we may state that the grounds of the hospital have an area of 176 acres; they present an uneven surface, the central parts rising 150 to 200 feet higher than at the entrance, with abrupt slopes near the boundaries to the east, south, and west. The grounds rise in irregular terraces, and the middle one of these has been chosen as the site of the main group of buildings, the chief of which faces south-west. The higher ground behind them is covered with an undergrowth of which affords protection from the north and east winds, and one half of the estate is heavily timbered woodland, especially towards the east and south. When these long slopes and picturesque terraces have a few lawn-openings and roads, a beautiful park will be made, with a cottage here and there and walks leading to quiet retreats under the great trees, with their curative influences of peacefulness and repose. The views from the grounds are extensive and beautiful. To the east is the City of Boston, with its harbour and neighbouring cities; to the south and south-west the village and famous Waverley oaks said to be four centuries old, form a pleasant picture; whilst further away to the south are the blue hills of Milton. Few more beautiful sites could have been chosen, and none more suitable for the McLean Hospital.

### Description of the McLean Hospital Buildings.

Entering the hospital grounds at the North Pleasant Street gate, nearest to the Waverley Station, which is seven miles from Boston, the visitor passes the Massachusetts General Hospital Convalescent Home on the left; if he takes the first turning to the right he will come to the house of the Superintendent, Dr. Cowles, which is pleasantly situated, and well isolated from the hospital buildings proper. If the visitor continues without turning to the right he finds himself in a short time at the Administration building, which stands on a terrace from which the Upham Memorial House, for nine men, is clearly visible in the grounds below. To the left when facing the Administration block, and a little behind it are the amusement hall, kitchens, and laboratory. Further to the left is Belknap House, behind which again is placed the women's gymnasium and Bowditch House, and to the left of Belknap is the Intermediate House for men.

The distance between these various houses is from 125 to 250 feet. To the right of the Administration building, when facing it, are placed the women's blocks, in the following order: Belknap House, having behind it the women's gymnasium, the pumping, heating, and electric stations, the Intermediate House for women, and Bowditch House for women; to the right of Belknap House, when facing it, are placed Appleton House, and away in the grounds, the house of the Medical Superintendent. One of the boundaries of the site is Mill Street, with an entrance gate, beyond which are the stables and greenhouses. In front of the Intermediate House for women is the Waverley spring, from which the water supply can be partly obtained, a supplementary supply having been arranged for with the Watertown Water Works. It is in contemplation to connect the drainage with the metropolitan sewer.

It will be seen that the main group consists of eight buildings for patients; they are connected as to their basements by a low covered way, so arranged as to appear from outside as an ordinary garden wall, about five feet high. It is in contemplation to erect some special hospital buildings, for a few acute cases of each sex, and it may be noted that the administration of the Upham Memorial House, which has no connecting corridor, will be largely independent. Generally it will be seen that each building has an individual and domestic character, unlike those of an institution—a character which is increased by varying the style of architecture and the materials used in construction. The plan provides for the effective separation of each household from its neighbours, a departure from the usual practice of placing such buildings near together for the sake of efficiency, ease, and economy of management, which constitutes the chief feature of interest in the plan.

Dr. Cowles holds that the welfare of patients as individuals requires that their residence shall be as home-like and free from harmful influences as possible. Proper organisation and management will meet the economic requirements, and the new order of nurses—the McLean Hospital is already famous for its nurse training school—their trustworthiness and efficiency, have rendered it possible for the trustees to make this most desirable improvement in hospital construction.

### The Eight Detached Houses.

The interior construction of all the buildings is alike

(2) Appleton House (McLean Hospital), 1st Floor Plan.

in many particulars that may be included in a general description. The main corridors and halls are enclosed by brick walls, with floors of brick, laid on guastavino arches. The partitions between the rooms, where not of brick, are made of terra-cotta blocks. All furring is of porous terra cotta, forming an air space within the wall, no white furring or lathing being used. All floors are deafened with plastering between the upper and under flooring. This construction tends to make the rooms sound proof and fire-proof. The stand pipes connected with the water tower are carried through each building, with outlets and hose on each floor. The corridors serve only as passages, and are not used as day-room space, but end in sitting-rooms of good size, almost all of which have "sunny corners." The corridors are lighted by alcoves, and there are open spaces over the staircases, which have easy flights, broad landings, and ornamental spindle-work. An attempt has been made throughout to secure domestic and home-like effects. The patients' rooms are so planned as to be capable of being used singly or *en suite*. All the sitting-rooms have fire-places, which are also provided in most of the buildings. The windows have large panes in the lower sashes.

On the women's side there are generally no guards to these windows, but simply screens of wire gauze. On the men's side the windows are treated somewhat differently for purposes of security where it is needed. The buildings are lighted throughout by electricity, and ornamental iron balconies, with awnings, form a feature in most of them. The lavatories, bath, and toilet rooms contain the most modern appliances, marble or tiles being used throughout.

Belknap and Appleton Houses for Women.

Belknap House has an attractive exterior of eastern red brick, laid with English bond, relieved by buff Amhurst stone facings. It is built in the shape of the letter L, with two entrances, the one in front facing south, the other at the eastern end of the south-east wing. This arrangement, with two staircases, divides the house into four distinct sections on each of the two floors, and each section has a lavatory and other necessary service rooms. The plan provides for the use of the rooms in small groups, for three or six patients, or in single isolated rooms. Nearly all the rooms face south-east or south-west, and are exposed to the sun at some time of the day. The building contains a fine entrance hall and dining-room, and the terraces which lead to the house add to its appearance. A good idea of the interior of this building will be obtained from the first floor plan (see p. 248). On the second floor the arrangement of the rooms is identical, except that over the supervisor's room and offices four rooms are placed for the partial isolation of special cases. The attics contain accommodation for twenty nurses, with a large and sunny sitting-room for their use. The basement contains kitchen, pantry, stores, and servants' sitting-room and other offices, and much of the cooking for the patients in each building will be done in its own kitchen, with a view to economy and the improvement of the service.

The Appleton building, which, like the Belknap, has been designed by Messrs. Fehmer and Page, is nearly square in shape. It is constructed of Bench brick, laid with Flemish bond, with white points, the window facings are of white marble, the under-pinning of cut granite, and the porches, cornices, dormers, &c., are of wood, the roof being slated. The main entrance has a large porch and *porte-cochère* leading to a spacious and well-lighted entrance hall and handsome staircase, with broad landings, ornamental railings and spindlework. As the accompanying first-floor plan shows, there is a reception-room to the right of the entrance, whilst facing this and at the end of the corridor are the serving and dining rooms. The house contains eight sets of rooms, each of which includes a sitting-room, bed-room, private lavatory, bath, and w.c. A thick wall running from the front to the rear of the building on both stories effectively isolates the eastern half of the house from the rooms near the main entrance. The porch is so planned as to be capable of being made into a "sun room" and conservatory during the winter. All the rooms are so planned as to permit each to be directly and easily inspected from the halls by the nurses, or each suite may be isolated, so that each chamber when occupied by a disturbed patient may be separated by an intervening lobby from the halls, and so be beyond the hearing of the other patients. The attics contain accommodation for a head nurse and sixteen nurses, whilst the kitchen, servants' offices, and stores are placed in the basement.

It would be wearisome to attempt a description in detail of each of the eight houses, but we have said enough to give a fair idea of the thought and care which has been expended upon the planning of the new McLean Hospital at Waverley, Mass., which will no doubt attract many visitors in the near future who desire to make themselves familiar with the most modern methods of asylum construction and treatment. We congratulate the trustees and Dr. Cowles upon the enterprise, knowledge, care, and judgment which have produced a set of plans for the new McLean Hospital which will no doubt attract the attention and excite the interest of experts everywhere.

## Figures and Tables

**Figure f1:**
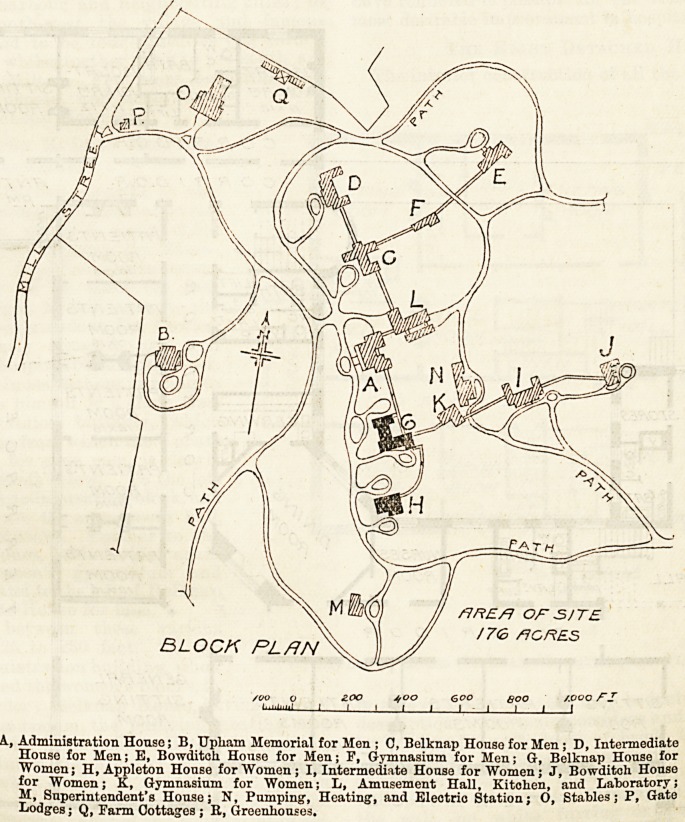


**Figure f2:**
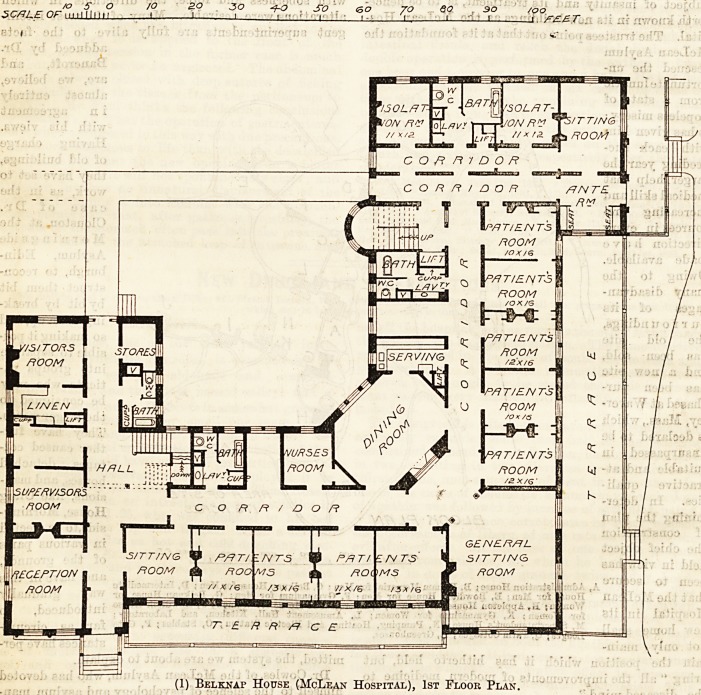


**Figure f3:**